# Chronic respiratory dysfunction due to diaphragmatic paralysis following penetrating neck trauma: A case report

**DOI:** 10.1097/MD.0000000000024043

**Published:** 2021-01-29

**Authors:** Lian Wang, Tianshu Liu, Zhihai Liu

**Affiliations:** a Department of Thoracic Surgery, the Second Affiliated Hospital, Zhejiang University School of Medicine; b Department of Critical Care Medicine, the First Affiliated Hospital, Zhejiang University School of Medicine, Zhejiang University, Hangzhou, China.

**Keywords:** diaphragm placation, diaphragmatic paralysis, penetrating neck trauma, phrenic nerve palsy

## Abstract

**Rationale:**

Respiratory dysfunction resulting from unilateral diaphragmatic paralysis during neck trauma is very rare in adults. We describe the symptoms, diagnosis and treatment of 1 patient with chronic respiratory insufficiency, in whom the diaphragmatic paralysis was associated with phrenic nerve injury due to penetrating neck trauma.

**Patient concerns:**

A 50-year-old worker was admitted because of left penetrating neck trauma. Imaging investigations demonstrated elevation of the left hemidiaphragm and the C5 and C6 roots avulsion. He complained of gradually worsening dyspnea on exertion 2 months later.

**Diagnoses:**

The patient was diagnosed with chronic respiratory dysfunction secondary to diaphragmatic paralysis, which caused by phrenic nerve injury.

**Interventions:**

A conventional video-assisted thoracoscopic diaphragm plication was performed after failed conservative management.

**Outcomes:**

The respiratory status improved markedly, and he did well without recurrence until 2 years’ follow-up.

**Lessons:**

The possibilities of phrenic nerve palsy and diaphragmatic paralysis should not be overlooked during the evaluation of neck trauma.

## Abstract 2

Supplemental Digital Content is available in the text

## 1 Introduction

Reports of phrenic nerve palsy and resultant diaphragmatic paralysis (DP) during penetrating neck trauma are uncommon. Most patients with unilateral DP might be asymptomatic, probably due to compensatory mechanisms.^[1]^ We describe 1 patient with chronic respiratory insufficiency in whom the DP was associated with phrenic nerve palsy due to penetrating neck trauma. A concurrent ipsilateral brachial plexus root avulsion was also identified and managed conservatively. Thoracoscopic diaphragm plication was performed and achieved excellent long-term improvements.

## 2 Case presentation

**Figure 1:**
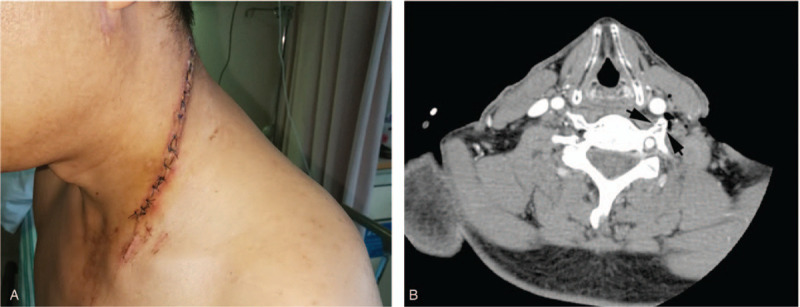
The left neck wound (A, day 4), and neck CT scan on admission showed a fracture of the transverse process of C5 (black arrows, B).

A 50-year-old worker was transferred to our emergency department due to a stab wound to the neck 1 hour before. On examination, there was a deep linear wound on the left side of neck (about 16 cm in length, Fig. 1A). He was a heavy smoker (2 packs a day). Contrast computed tomography showed penetrating foreign bodies, soft tissue defects in the lateral neck, and left transverse process fractures of C5 and C6 (Fig. 1B). We performed wound debridement and inserted a surgical drain system.

**Figure 2:**
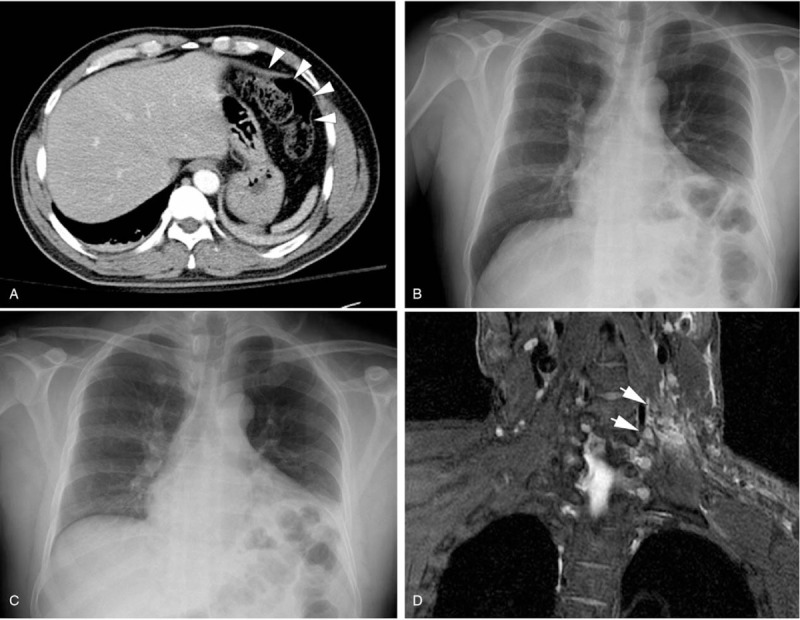
(A). Abdominal CT scan demonstrated displacement of colon to left upper quadrant (white arrowheads). (B and C). Paired inspiratory and expiratory CXRs. (D). Coronal T2-weighted MRI showed hyperintensity (white arrows) of C5-C6 nerve roots. MRI = magnetic resonance imaging.

However, the patient complained of pain in his upper abdomen followed by nausea and vomiting on the 5th day, when he was supposed to be discharged. The abdominal computed tomography revealed an unusually elevated left hemi-diaphragm with displacement of colon to left upper quadrant (Fig. 2A). This was not evident on a chest x-ray (CXR) taken 4 months previously. Ultrasound of chest detected a thin diaphragm with nearly no diaphragmatic movement in the left side during deep breathing (Supplemental Video (Video that demonstrates the normal right diaphragmatic excursion and contraction during deep inspiration, but no diaphragmatic movement in the left side, 1 minute, 33MB), http://links.lww.com/MD/F569). Inspiratory/expiratory CXRs revealed little movement of the left diaphragm (Fig. 2B and C), raising suspicion of DP secondary to phrenic nerve palsy. Furthermore, the brachial plexus magnetic resonance imaging showed left C5 and C6 root avulsion injuries (Fig. 2D). A diagnosis of brachial plexus injury, occurring concurrently with left hemidiaphragmatic paralysis caused by phrenic nerve palsy was made. The subsequent pulmonary function test (PFT) showed significant ventilatory impairment, forced vital capacity (FVC) 2.28L (61% of predicated value), and forced expiratory volume in 1s 65L (53% of predicated value) with a mild diffusion defect. The patient had no shortness of breath and was discharged on day 7 with oral mecobalamin.

Two month later, CXR showed a markedly elevated left diaphragm with associated left lower lobe atelectasis. A repeated PFT revealed FVC 1.83L (49% of predicated value), and forced expiratory volume in 1s 1.34L (43% of predicated value). On supine position the FVC further decreased to 1.4L. During the next year the patient experienced progressively increasing shortness of breath and dyspnea when climbing stairs. His performance status deteriorated to a Medical Research Council Dyspnea Scale grade of at least 2. Therefore, he was diagnosed with chronic respiratory insufficiency secondary to DP. The patient underwent a multiportal video-assisted thoracic surgery (VATS) diaphragm plication using a single running suture technique. He was well recovered and discharged on the 3rd day after surgery.

The post-operative CXR showed the elevated diaphragm was generally reduced and the adjacent atelectasis had completely resolved. The respiratory status improved markedly after surgery, and he did well without recurrence until 2 years’ follow-up.

## 3 Discussion

We presented a case of DP secondary to phrenic nerve palsy with concurrent brachial plexus injury which occurred after penetrating neck trauma. The patient developed a gradual worsening of respiratory symptom that was successfully treated by thoracoscopic diaphragm plication.

Because of the high concentration of vital structures in a small anatomic area, penetrating neck injuries are associated with substantial morbidity and mortality.^[2]^ As a result, the Advanced Trauma Life Support guidelines recommend that vessels and airway injuries should be identified first during the early stages of the evaluation. However, damage to the pharyngeal, oesophageal, spinal cord and nerves are also very common.^[2]^ Although this patient was incredibly fortunate that there was no life-threatening bleeding or tracheal rupture, he presented with phrenic nerve palsy, which resulted in DP.

The diaphragm generates 80% of the lung volume change during quiet inspirations.^[3]^ The innervation of the diaphragm comes exclusively from the phrenic nerve. The latter originates from the anterior rami of C3 through C5 nerve roots, while the brachial plexus receives contributions from spinal nerves C5 to T1. Because of the proximity of these closely adjacent structures, phrenic nerve injury and hemidiaphragmatic paralysis commonly occur in association with brachial plexus trauma. Magnetic resonance imaging provides accurate anatomic and physiologic information on brachial plexus injury.^[4]^ The estimated incidence of an associated phrenic nerve palsy with brachial plexus injury ranges from 10% to 20%,^[5]^ while traumatic injury to the phrenic nerve is the most common cause of unilateral diaphragmatic weakness.^[3]^ Therefore, 1 should always consider the possibility of concurrent ipsilateral phrenic nerve palsy when diagnosing a patient with brachial plexus injury.

An abnormally elevated hemidiaphragm is often found accidentally on CXR. The fluoroscopic sniff test is traditionally a first line and real time fluoroscopic evaluation of diaphragmatic dysfunction but false negative results could not be excluded.^[6]^ In most cases, a combination of imaging investigations along with specific clinical conditions can be useful to assess diaphragmatic dysfunction.^[7]^ Ultrasonography, a radiation-free and simple bedside tool, has recently become increasingly popular in the assessment of diaphragm in various clinical conditions.^[8]^ In patients with unilateral paralysis, the paralyzed side shows reduced diaphragmatic thickening and no (or even paradoxical) movement during inspiration. PFT in both sitting and supine position is also recommended for suspected diaphragmatic palsy. The characteristic feature on the PFT is restrictive ventilatory dysfunction. As diaphragmatic electromyography and transdiaphragmatic pressure measurement require specialized equipment and expertise for interpretation, they are not recommended for routine evaluation of DP.^[3]^

Most unilateral DP are asymptomatic during quiet breathing,^[9]^ but may result in breathlessness or dyspnea on exertion. In this case he began getting short of breath at 2 months after phrenic nerve palsy, followed by progressive exertional dyspnea. A history of heavy smoking undoubtedly exacerbated the lung function loss attributable to DP.

Correspondingly, conservative management is the primary option for most unilateral diaphragmatic dysfunction. Correction of unilateral diaphragm paralysis by diaphragm placation is a relatively infrequent surgery, but this is a well employed method that substantially improves dyspnea in patients whose symptoms last for more than a year.^[3]^ Plication increases the hemithorax volume, and reduces lung compression and the paradoxical motion. Diaphragmatic plication can be performed through both transthoracic and transabdominal approaches. Sezai et al reported a small series study with 12 patients with diaphragm paralysis, and observed that diaphragm plication by thoracotomy significantly improves pulmonary function in long-term follow-up.^[10]^ VATS diaphragm plication minimizes surgical trauma and potentially decreases postoperative morbidity and hospitalization. Uniportal video-assisted and robotic-assisted thoracoscopic diaphragm plication have also been reported recently for symptomatic diaphragm paralysis.^[11,12]^

## 4 Conclusion

In the evaluation of neck trauma, it is important to consider the possibilities of brachial plexus root avulsion, concomitant phrenic nerve injury as well as ipsilateral DP. Minimally invasive thoracoscopic diaphragm placation remains a viable option in the management of patients suffering from chronic respiratory impairment due to DP.

## Acknowledgment

Thanks for this patient who consented to publish his case.

## Author contributions

**Conceptualization:** Zhihai Liu.

**Funding acquisition:** Lian Wang.

**Investigation:** Zhihai Liu, Lian Wang, Tianshu Liu.

**Writing – original draft:** Lian Wang, Tianshu Liu.

**Writing – review & editing:** Zhihai Liu.

## Abbreviations

Abbreviations: CXR = chest x-ray, DP = diaphragmatic paralysis, FVC = forced vital capacity, PFT = pulmonary function test.
